# Acupuncture for primary osteoporosis: A network meta-analysis of randomized controlled trials protocol

**DOI:** 10.1097/MD.0000000000015108

**Published:** 2019-04-12

**Authors:** Fan Huang, Siyi Zhao, Mingwang Qiu, Yitong Li, Xiaoxuan Zhan, Cunshu Wu, Chushuo Shi, Weipeng Sun, Guizhen Chen, Yunxiang Xu

**Affiliations:** a Clinical Medical College of Acupuncture, Moxibustion and Rehabilitation; b The First Medical College, Guangzhou University of Chinese Medicine, Guangzhou; c Shenzhen Bao’an Traditional Chinese Medicine Hospital, Guangzhou University of Chinese Medicine, Shenzhen, Guangdong, China.

**Keywords:** acupuncture, network meta-analysis, primary osteoporosis, protocol, systematic review

## Abstract

**Background:**

A large number of randomized controlled trials (RCTs) have shown that acupuncture (ACU) has certain advantages over pharmacotherapies in the treatment of primary osteoporosis (POP). However, due to the diversity of ACU treatments, its relative effectiveness have not yet been studied and explained. Therefore, based on the network meta-analysis (NMA), this study will compare the differences in the efficacy of multiacupuncture in the treatment of POP, to provide a reference for clinical treatment.

**Methods:**

We will search PubMed, MEDLINE, Embase, the Cochrane Library, China National Knowledge Infrastructure (CHKD-CNKI), WANFANG database (Chinese Medicine Premier), Chinese Biomedical Literature database (CBM), and VIP for relevant RCTs of ACU treatments for POP, from their inceptions to January 2019. STATA 15.0 and GEMTC software will be used to perform a NMA. The evidence will be evaluated by the Grading of Recommendations Assessment, Development, and Evaluation (GRADE) approach and the type 1 error rate will be assessed by trial sequential analysis (TSA).

**Results:**

The results of this review will be submitted to a recognized journal for publication.

**Conclusion:**

This proposed systematic review will evaluate the different advantages of various types of ACU in the treatment of POP.

**Registration:**

PROSPERO (registration number CRD42019122724).

## 1 Introduction

Primary osteoporosis (POP) is a systemic degenerative disease of skeletal physiology, which is caused by abnormal metabolism of the skeleton itself, resulting in decreased bone strength and increased risk of fracture.^[1]^ With the advent of an aging society, the incidence of POP at home and abroad is gradually increasing, and its common symptoms include pain, spinal deformity, and fragile fracture, and that is one of the main causes of disability and death of elderly patients.^[2]^ Moreover, medical treatment and care for osteoporosis will impose a heavy financial burden on families and society. The annual medical cost of fracture in the United States is between $10 billion and $17 billion, and the total cost is expected to be $25.3 billion by 2025.^[3]^

At present, there is a lot of drugs for the treatment of POP in clinic, mainly including the broad-spectrum antifracture drugs and injection preparations, such as alendronic acid, zoledronic acid, risedronate sodiu, teriparatide, and so on.^[4,5]^ Although these drugs have certain clinical curative effect,^[3,6]^ long-term use of them will have many side effects (such as a typical femoral subtrochanteric fractures, gastrointestinal side effects, hot flashes, thromboembolism events, and some drug-specific infections).^[7,8]^ Even if there is evidence that drugs, for instance, raloxifene and risedronate can reduce the probability of vertebral fracture, some studies have shown these drugs cannot reduce all types of fractures, and that patients with fragile fractures who rely solely on drugs treatment will have a fivefold risk of fracture in the first 2 years after fracture.^[9]^

In terms of traditional Chinese medicine, the analgesic effect of acupuncture (ACU) has been internationally recognized.^[10,11]^ Cellular mechanism underlying ACU-induced analgesia has been suggested to be the purinergic signaling system works through interactions with different purine receptors.^[12,13]^ Recent studies have shown that warm ACU and electroacupuncture as special ACU can improve clinical symptoms and relieve pain of osteoporosis. ACU inhibits subchondral bone loss by regulating the signaling pathway of bone protection protein (OPG)/nuclear factor-K β receptor activator (RANK)/nuclear factor-K β receptor activator ligand (RANKL), and reduces the expression of cathepsin mRNA in rats.^[14–17]^ In addition, compared with other treatments, ACU has significant advantages in terms of overcoming the high cost of health care and reducing the side effect of drugs.

In the study of POP, because bone remodeling is sustained for approximately 180 to 190 days in basic multicellular units dependent on mechanical micro-injury,^[18]^ using RCT for clinical effect verification may need the long duration of time, costly capital, and lots of manpower and resources. At present, there are many kinds of ACU for the treatment of POP, but various ACU have their characteristics and effectiveness. Therefore, to optimize ACU treatment of POP, this study will compare the characteristics of various ACU of NMA to provide a foundational reference for clinical treatment.

## 2 Methods

### 2.1 Study registration

The protocol has been registered on PROSPERO CRD42019122724 (https://www.crd.york.ac.uk/PROSPERO/).

### 2.2 Eligibility criteria

#### 2.2.1 Type of study

We will include all RCTs of ACU for POP, regardless of allocation concealment, or used of blinding. The language of the literature will be limited to Chinese or English.

#### 2.2.2 Participants

All patients have POP (as diagnosed using any recognized diagnostic criteria) and must be over 18, any sex, disease stage, or severity.

#### 2.2.3 Interventions

The experimental group must be treated with ACU, including electroacupuncture, acupoint catgut embedding, auricular acupoint stimulation, or fire ACU. Whether applied alone or in combination with pharmacotherapies. The control group is treated with non-ACU or pharmacotherapies. Pharmacotherapies include drugs recommended in international or domestic authorized clinical guidelines. Studies which combine ACU treatments with pharmacotherapy are required to use the same pharmacotherapy in both the experimental and the control groups.

#### 2.2.4 Outcomes

The primary outcomes are efficacy, visual analogue scales (VAS), and bone mineral density (BMD). The secondary outcomes are blood alkaline phosphatase (ALP) and adverse reactions.

### 2.3 Search strategy

We will search the following electronic bibliographic databases: PubMed, MEDLINE, Embase, the Cochrane Library, China National Knowledge Infrastructure (CHKD-CNKI), Wanfang database (Chinese Medicine Premier), Chinese Biomedical Literature database (CBM), and VIP. All of them will be searched from inception to January 2019. And we will perform the search strategies outlined in the Cochrane Handbook for Systematic Reviews of Interventions Version 5.1.0.^[19]^ Searching terms will be used as MeSH terms and free-text.

### 2.4 Study selection and data extraction

Two reviewers (HF and ZSY) will independently conduct literature screening, data extraction, and check. In case of disagreements, a third reviewer will participate in consensus conferences. In the literature screening, the titles and abstracts of retrieved studies should be read first, and the full-text of the included studies will be read after excluding irrelevant literature to determine whether to include or not. The following information will be extracted from the included trials using a PRISMA flowchart and Excel 2010: author, year of publication, country, study design, sample size, participants, ACU intervention, control intervention, outcomes, and adverse events. We will use the PRISMA flowchart to represent the complete process (available for download at http://www.prisma-statement.org).

### 2.5 Risk of bias assessment

We will independently assess risk of bias for each selected study in accordance with the Cochrane Handbook 5.1.0 for Systematic Reviews of Interventions, which comprises 7 items: random sequence generation, allocation concealment, blinding of participants and personnel, blinding of outcome assessment, incomplete outcome data, selective reporting, and other sources of bias. The studies will be evaluated as being of “low risk of bias,” “high risk of bias,” or “unclear risk of bias.”^[20]^ At the same time, the included clinical trials will be awarded a score from 0 to 5 points according to Jadad scale evaluation criteria, which include reference to the generation of random sequences, blind enforcement, and withdrawal.^[21]^

### 2.6 Statistical analysis

#### 2.6.1 Pairwise meta-analysis

We will perform pairwise meta-analyses of direct evidence using the random-effects model with STATA 15.0 software (STATA Corp, College Station, TX), whereas a random-effects NMA within a Bayesian framework will be performed. Where different measures are used to assess the same outcome, dichotomous outcomes data will be analyzed by calculating Mantel–Haenszel odds ratios (ORs) and continuous outcomes will be pooled with standardized mean difference (SMD). We will present 95% confidence intervals for all outcomes. The data will be managed on an intention-to-treat (ITT) basis as far as possible. Attempts will be made to obtain missing data from the trial authors. Where data are unobtainable, we will assume that an event, without a reported outcome, has not occurred in participants and we will analyze only the available data.

#### 2.6.2 Network meta-analysis

STATA 15.0 and GEMTC software (Generate Mixed Treatment Comparisons, http://drugis.org/gemtc-gui) will be used to perform NMA to compare direct and indirect evidence. After comparing multiple interventions, we will calculate the surface under the cumulative ranking curve (SUCRA); the SUCRA values will be used to rank the pros and cons of interventions. TSA 0.9 (Copenhagen Trial Unit, Copenhagen, Denmark) will be used to perform trial sequential analysis (TSA) for assessing the type 1 error rate for selected pooled estimates.^[22]^

#### 2.6.3 Measures for inconsistency

For a closed loop of 3 treatments, the inconsistency between direct and indirect evidence will be directly examined. For the closed loop formed by the 4 studies, it can be divided into 2 closed triangular loops to test the inconsistency between direct evidence and indirect evidence. In every closed loop, we will use “Inconsistency Factors” value (IF) to calculate the absolute difference; when the “Inconsistency Factors” value is close to 0 and its 95% CI crosses 0, this indicates that the degree of inconsistency is low and the consistency model will be selected. On the contrary, when the “Inconsistency Factors” value is far from 0 and its 95% CI does not contain 0, the degree of inconsistency is larger and the inconsistency model will be selected.

#### 2.6.4 Quality of evidence

The quality of evidence will be assessed with the GRADE approach.^[23]^

## 3 Discussion

Systematic review has shown the effectiveness and safety of ACU for the treatment of POP, but it lacks studies which directly compare among different ACU methods, so that clinicians cannot judge the therapeutic value of different forms of ACU, which will be not conducive to choose the best ACU treatment. Therefore, NMA will be used to compare the difference in effectiveness among various ACU methods, so as to provide reliable evidence-based medical evidence for clinical promotion and efficacy evaluation of ACU treatments for POP. This protocol has been registered with international prospective register of systematic reviews (PROSPERO), and it will be executed strictly in accordance with the NMA steps. In addition, the quality of evidence for the main outcomes will be assessed with the GRADE approach. The NMA will be based on the PRISMA extension statement for reporting of systematic reviews incorporating network meta-analyses of healthcare interventions. However, only English and Chinese literatures will be included in this study, which will be lead to selection bias. And most of the included literatures have some differences in ACU treatment time and acupoint selection, which may have certain effects on the curative effect, so we will use subgroup analysis to reduce the inconsistency. In summary, we hope this study can provide more evidence sources for ACU in the treatment of POP.

## Author contributions

**Data curation:** Cunshu Wu.

**Formal analysis:** Fan Huang.

**Funding acquisition:** Yunxiang Xu.

**Methodology:** Chushuo Shi.

**Project administration:** Guizhen Chen.

**Validation:** Weipeng Sun.

**Writing – original draft:** Mingwang Qiu, Xiaoxuan Zhan.

**Writing – review and editing:** Fan Huang, Siyi Zhao, Yitong Li.
