# Acupuncture for primary osteoporosis: A network meta-analysis of randomized controlled trials protocol: Erratum

**DOI:** 10.1097/MD.0000000000015898

**Published:** 2019-05-24

In the article, “Acupuncture for primary osteoporosis: A network meta-analysis of randomized controlled trials protocol”,^[1]^ which appeared in Volume 98, Issue 15 of *Medicine*, the second corresponding author's information is missing and should appear as Yunxiang Xu, No. 232, Waihuan East Road, Guangzhou 510405, Guangdong, China (e-mail: xuyx1968@163.com). The first corresponding author's, Guizhen Chen, email should be cgzhen2000@163.com.
